# Immunotherapy: incorporation in the evolving paradigm of renal cancer management and future prospects

**DOI:** 10.18632/oncotarget.14388

**Published:** 2016-12-30

**Authors:** Kenneth G. Liu, Sorab Gupta, Sanjay Goel

**Affiliations:** ^1^ Department of Medical Oncology, Montefiore Medical Center, Bronx, NY, USA; ^2^ Department of Internal Medicine, St. Barnabas Hospital, Bronx, NY, USA

**Keywords:** renal cell carcinoma, immunotherapy, checkpoint Inhibition, cytokines, vaccines

## Abstract

Significant progress has been made in the management of renal cell carcinoma (RCC) during the last few decades. In early stage, localized disease, surgical resection remains the modality of choice, with no therapeutic interventions as options for post-operative therapy other than simple observation and clinical surveillance. However, treatment options in the advanced or metastatic setting are increasing at a dizzying pace, initially with cytokine therapy, then with the increased availability of targeted therapy including novel small-molecule inhibitors of receptor tyrosine kinases and monoclonal antibodies targeting novel proteins, establishing them as the current standard of care. Even more recently, immunotherapy has seen tremendous development in the form of immune checkpoint inhibition and vaccines. Overall, these interventions have gradually changed the landscape of cancer management in general, and metastatic renal cell carcinoma (mRCC) in particular. This is exemplified by the recent United States Food and Drug Administration (USFDA) approval of nivolumab for patients with mRCC after failure of TKI therapy. In this review, we present a brief overview of the current management of mRCC, primarily the clear cell subtype (ccRCC), and discuss the major clinical trials and data on the immunotherapy in advanced or mRCC.

## INTRODUCTION

For decades, therapeutic modalities for cancer management have primarily consisted of surgery, radiotherapy and chemotherapy. In renal cell carcinoma (RCC), surgical resection, either *via* nephron-sparing or radical nephrectomy, can be curative in patients with early stage, localized disease. However, no adjuvant treatment has been proven to be beneficial. A significant proportion of patients eventually develop tumor recurrence, and subsequent therapeutic options in the advanced or metastatic setting are limited. Since the turn of the millennium, novel small-molecule targeted therapy has evolved to become the mainstay of treatment for metastatic renal cell carcinoma (mRCC), though prognosis remains poor [1]. The past decade has seen the emergence of immunotherapy as an exciting treatment option for various malignancies, including RCC. The most common forms of immunotherapy include cytokine therapy and immune checkpoint inhibition, although vaccines are also being investigated. Cytokine therapy such as IL-2 and IFN-α was commonly used in the 1990’s for mRCC, though its use has declined given modest response rates and poor tolerability. On the other hand, immune checkpoint inhibition has made significant progress and gained much attention with the approval for use in various solid tumors such as melanoma, non-small cell lung cancer and more recently, RCC [1, 2]. In this review, we present a brief overview of the current management of mRCC, primarily the clear cell subtype (ccRCC), and discuss the major clinical trials and data on the immunotherapy in advanced or mRCC.

## EPIDEMIOLOGY

RCC represents 2-3% of all cancers, with the West contributing its major share to the associated morbidity and mortality. Worldwide, 209,000 new cases and 102,000 deaths per year are attributed to RCC [3]. RCC comprises 90% of all primary renal neoplasms and is a heterogeneous disease that progresses along varied pathophysiological pathways. The most common subtypes are clear cell, papillary and chromophobe, while there are at least ten other rarer subtypes based on recent re-classification by the International Society of Urologic Pathology [4]. RCC is known to occur predominantly in the sixth and seventh decades of life [3]. Young adults less than 40 years of age and children are rarely affected, though they are more likely to have symptomatic tumors [5, 6]. It is also known to have a male predilection as compared to females (2:1), making it the seventh most common cancer in men while being the ninth most common cancer in women [3]. The incidence of RCC differs among various ethnic populations. In the United States, incidence rates are lowest among Asian and Pacific Islanders, while being much higher among Whites and Blacks, suggesting a potential role for both genetic and environmental factors [7]. The risk of developing RCC may be increased by lifestyle-modifying factors such as smoking (both active and passive), obesity and hypertension. It is also more common in patients with end-stage renal disease, acquired renal cystic disease, and tuberous sclerosis. In addition, environmental exposures to asbestos, cadmium, dry-cleaning solvents, gasoline and other petroleum by-products, as well as prolonged use of non-aspirin NSAIDs may increase the risk of developing RCC [8, 9]. Approximately 2-3% of RCC are familial, with von Hippel-Lindau syndrome being the most common [3].

## PROGNOSIS

Most RCCs are clinically silent during their course and therefore a diagnosis may not be made until they become locally advanced or metastatic. However, in more recent years, a significant proportion of RCCs are also detected incidentally. The difference in prognosis between early and advanced stage disease is striking. In the United States, the 5-year survival (from 2005-2011) was 92% for localized disease, 65% for regional disease (spread to the lymph nodes), and only 12% for distant disease (metastases) [10]. These statistics not only highlight the importance of developing effective therapy in the metastatic setting, but also lead to the development of different prognostic models to help guide treatment in advanced RCC.

The UCLA Integrated Staging System (UISS) is a prognostic model that incorporates information obtained from anatomic stage (based on the 1997 tumor-node-metastasis stage), Fuhrman’s grade, and the Eastern Cooperative Oncology Group (ECOG) performance status. Based on these criteria, patients were divided into low, intermediate and high risk categories. This system was validated in a prospective cohort involving RCC patients who underwent nephrectomy [11, 12]. For recurrent or mRCC, one of the most commonly used prognostication systems is the one proposed by the Memorial Sloan Kettering Cancer Center (MSKCC) group that integrates five adverse factors: low Karnofsky performance status score (less than 80), high serum lactate dehydrogenase level (LDH; greater than 1.5 times the upper limit of normal), low hemoglobin level (less than the lower limit of normal), high corrected calcium (greater than the upper limit of normal), and short interval from diagnosis to treatment (less than one year). In the original study, patients with mRCC received interferon-α as first-line therapy. Patients with none of the risk factors compared to those with one or two and those with three or more risk factors had significantly higher one-year (83% *vs*. 58% *vs*. 20%) and three-year overall survival (OS) rates (45% *vs*. 17% *vs*. 2%) [13]. In the era of targeted therapy, the International Metastatic RCC Database Consortium (IMDC) model has been proposed. The IMDC model is based on six adverse clinical factors, four of which are from the MSKCC model (Karnofsky performance status, diagnosis-to-treatment interval, hemoglobin, and corrected calcium). In addition, neutrophils and platelets greater than the upper limit of normal were identified as independent adverse prognostic factors. This model was validated in a large, multicenter study involving mRCC patients who received sunitinib, sorafenib and bevacizumab [14].

## CURRENT TARGETED THERAPY

Currently, targeted therapy involving tyrosine-kinase inhibitors (TKI), especially, the anti-vascular endothelial growth factor (VEGF) agents are widely used in the first and second line treatment for mRCC. Prognostic risk stratification as described earlier is often helpful in the selection of the targeted therapy. As ccRCC represents the most common subtype of RCC, it has been the major focus of clinical trials. In the front-line setting, sunitinib, pazopanib and temsirolimus are the agents most routinely used based on data from a number of clinical trials. Sunitinib, a multikinase inhibitor, was compared against IFN-α in the first-line setting and was found to have improved progression-free survival (PFS) and OS. The vast majority of the patients in the trial were considered “favorable” or “intermediate” according to the MSKCC prognostic model, therefore, sunitinib is typically used in patients meeting such criteria [15, 16]. Pazopanib is another multikinase inhibitor that has shown efficacy in the first-line setting. Pazopanib was compared to placebo and was shown to have significantly prolonged PFS [17]. OS was similar, but could be explained by the extensive crossover of placebo patients onto the pazopanib arm [18]. Pazopanib was also directly compared with sunitinib, with pazopanib showing similar efficacy but appeared to be better tolerated than sunitinib [19, 20]. Meanwhile, temsirolimus, an inhibitor of the mammalian target of rapamycin (mTOR) protein, has been investigated in “poor” risk patients. In a randomized phase III trial, temsirolimus was evaluated as monotherapy or in combination with IFN-α. Temsirolimus was found to have improved PFS and OS compared to IFN-α, while the combination did not yield additional benefit despite an increase in adverse events [21]. Based on these data, temsirolimus is now approved for first-line treatment of “poor” risk metastatic RCC. Other acceptable treatment options in the front-line setting include bevacizumab (an anti-VEGF monoclonal antibody) in combination with IFN-α [22, 23], sorafenib (a multikinase inhibitor) [24] and axitinib (a multikinase inhibitor that acts by inhibiting VEGF) [25].

For therapy in the second-line setting and beyond, the choice of targeted agent is typically tailored based on what the patient receive in the first-line setting. Cabozantinib, another multikinase inhibitor, was studied with everolimus as the control, in patients who had been treated with prior TKI therapy, and was shown to have improved PFS and OS [26, 27]. Axitinib can be used in patients who received one prior systemic therapy based on a randomized trial where it was compared with sorafenib and was shown to have improved PFS but not OS [28, 29]. Meanwhile, lenvatinib alone and in combination with everolimus were found to prolong PFS when compared to everolimus alone for patients who have received one prior anti-angiogenic therapy, with the combination recently being approved by the United States Food and Drug Administration (USFDA) [30]. Finally, everolimus alone [31], sorafenib [32] and pazopanib [17] have all been investigated in the second-line setting when compared to placebo and demonstrated improved PFS. Other agents that are available include sunitinib, temsirolimus and bevacizumab.

In summary, we believe that the management of mRCC has turned into an “embarrassment of riches” with a multitude of options, and it is almost impossible that a patient will be able to avail of all the options available. Our recommendation when dealing with a newly diagnosed mRCC patient is to first perform risk stratification and determine prognosis. For patients with good or intermediate risk and ccRCC, offer sunitinib or pazopanib as these appear to have the best data, and can be used as a single agent. For patients with poor risk or non clear cell RCC, offer temsirolimus as front line therapy. For patients who are intolerant to one of these agents, offer them an alternate therapy from among the front line agents listed above. For those patients who have progressed on front line therapy, the preferred options are nivolumab, cabozantinib, or the combination of lenvatinib and everolimus, as these three options have demonstrated a survival advantage.

## CYTOREDUCTIVE NEPHRECTOMY

Prior to the widespread use of targeted therapy and immunotherapy, mRCC was associated with a dismal prognosis given the limited role of chemotherapy. Treatment was limited to cytokine therapy. Cytoreductive nephrectomy was proposed as a modality to improve outcomes. This was based on several reports in the literature suggesting spontaneous regression of metastases in patients who have undergone nephrectomy [33, 34]. While the exact mechanism is not entirely known, immunologic factors are felt to play important role [35]. Cytoreductive nephrectomy was subsequently investigated in two similar randomized phase III trials. In a study conducted by the Southwest Oncology Group (SWOG), patients were randomized to receive either radical nephrectomy followed by IFN-α-2b, or IFN-α-2b therapy alone. OS was 11.1 months in the surgery arm compared with 8.1 months in the interferon-only arm (*P* = 0.05) [36]. Meanwhile, the European Organisation for Research and Treatment of Cancer (EORTC) similarly compared radical nephrectomy followed by IFN-α *versus* IFN-α alone. OS was significantly improved (17 months *vs*. 7 months) favoring the surgery arm [37]. However, cytoreductive nephrectomy is not without its morbidity, and careful selection of patients should be undertaken. A retrospective analysis identified 7 independent preoperative predictors of inferior OS in surgical patients: a LDH level greater than the upper limit of normal, an albumin level less than the lower limit of normal, symptoms at presentation caused by a metastatic site, liver metastasis, retroperitoneal adenopathy, supradiaphragmatic adenopathy, and clinical tumor classification ≥ T3. Patients with ≥ 4 risk factors did not appear to benefit from surgery [38]. Finally, the role of cytoreductive nephrectomy is not entirely clear in patients who will undergo targeted therapy. A study published by the International Metastatic RCC Database Consortium demonstrated improved OS for patients who underwent cytoreductive nephrectomy who are subsequently treated with VEGF-targeted agents, although the benefit is marginal in those with poor risk features [39]. A randomized phase III trial (CARMENA) is currently underway to investigate the importance of cytoreductive nephrectomy in mRCC patients treated with sunitinib [NCT00930033] [40].

## IMMUNOTHERAPY

Immunotherapy is often considered a new treatment modality in the management of cancer. However, its use was first reported back in the late 19th century, when surgeon William Coley demonstrated that the injection of killed bacterial products into inoperable sarcoma tissue led to the shrinkage of tumor [41]. He subsequently developed a mixed bacterial vaccine and was able to achieve long-term remissions in some patients with sarcoma and various tumor types [42, 43]. It was later recognized that cancer cells express tumor antigens that may stimulate cellular and/or humoral responses. Peptides derived from tumor antigens are presented *via* major histocompatibility complex (MHC) class I and class II epitopes and may stimulate CD8+ and CD4+ T cells respectively [2]. The binding of the T cell receptor (TCR) to the peptide presented by MHC requires further co-stimulatory signals, and its interaction activates downstream pathways resulting in the secretion of proinflammatory cytokines [44]. However, the amplitude and quality of the response are regulated by a balance between co-stimulatory and inhibitory signals, known as immune checkpoints [45].

In mRCC, various forms of immunotherapy, including cytokines, immune checkpoint inhibitors, vaccines, have been used or tested. The following sections will discuss these in more detail.

### Cytokine therapy

Prior to the development of targeted therapy, cytokines such as IL-2 and IFN-α were used for the management of mRCC. However, these are typically associated with significant toxicity with mild clinical benefit. High dose IL-2 was approved by the USFDA as early as 1992 for the treatment of mRCC based on data from phase II trials. Fyfe et al. reported data on 255 patients (from 7 different phase II trials) who received high dose IL-2. The objective response rate (ORR) was 14% (5% complete response and 9% partial response). However, treatment was associated with significant toxicities, including grade 3 and 4 hypotension (74%), nausea/vomiting (25%), diarrhea (22%), mental status changes (28%), elevated bilirubin (21%), oliguria/anuria (46%), fever/chills (24%), thrombocytopenia (21%), though recovery was considered rapid [46]. A subsequent retrospective analysis reviewed data from 259 mRCC patients treated with high dose IL-2 at the National Cancer Institute between 1986 and 2006, and confirmed a 20% ORR. Of note, disease recurrence occurred in all patients with partial response (PR), while 19 of 23 patients (83%) with complete response (CR) were disease-free at the time of last follow-up [47].

Further studies focused on determining whether lower doses of IL-2 could produce similar efficacy with lower toxicity. The Cytokine Working Group conducted a randomized phase III study which compared high dose IL-2 with the outpatient combination of IL-2 and IFN-α. The response rate was 23.2% in the high dose IL-2 group *versus* 9.9% in the outpatient combination group. No significant differences in PFS and OS were noted. However, significant difference in OS favoring high dose IL-2 were seen in patients with liver and bone metastases (*P* = 0.001) and in patients whose primary tumors were still in place (*P* = 0.040) [48]. The National Cancer Institute designed a phase III trial comparing high-dose IL-2 (720,000 U/kg every 8 hours) and low-dose IL-2 (72,000 U/kg every 8 hours). Toxicities were less frequent with low-dose IL-2, but there was a higher ORR with high-dose (21%) compared with low-dose (13%). No OS difference was noted [49]. Based on results from these studies, high-dose IL-2 can potentially achieve durable responses in carefully selected patients with mRCC. However, it has gradually fallen out of favor in the first-line setting in this current era of targeted therapy and immunotherapy.

### Checkpoint inhibition

There are a number of mechanisms through which immune invasion by tumor cells can occur. These mechanisms include downmodulation of tumor antigen presentation by downregulation of MHC class I molecules, upregulation of inhibitors of apoptosis, or expression of cell surface molecules that directly kill cytotoxic T cells. Tumors may also release factors that induce inhibition of both the innate and the adaptive anti-tumor immunity, as well as recruit regulatory cells to generate an immunosuppressive microenvironment [50, 51]. Finally, immune checkpoint proteins may become dysregulated, typically *via* overexpression of inhibitory ligands and receptors that regulate T cell effector functions in the tumor microenvironment [45].

The two immune checkpoint receptors most commonly targeted are the cytotoxic T-lymphocyte-associated antigen 4 (CTLA-4) and programmed cell death protein 1 (PD-1). CTLA-4 is present on the surface of T cells and counteracts the action of co-stimulatory receptor CD28. Both CTLA-4 and CD28 bind identical ligands CD80 and CD86, but CTLA-4 does so at a higher affinity, thus out competing CD28 and dampens the activation of T cells. In addition, CTLA-4 may directly sequester CD80 and CD86 from CD28 engagement, as well as active removal of CD80 and CD86 from the antigen-presenting cell surface [45, 52]. CTLA-4 antibodies were initially tested on mouse models of colon adenocarcinoma and sarcoma and were noted to induce tumor shrinkage [53]. These encouraging results subsequently led to the development of two CTLA-4 antibodies, ipilimumab and tremelimumab.

PD-1 is a transmembrane protein that is more broadly expressed than CTLA-4. It is found in T cells, as well as B cells and natural killer cells. It binds to two ligands, PD-L1 and PD-L2, which are commonly expressed on the tumor cell surface of multiple tumor types. The interaction of PD-1 and its ligands inhibits kinases that are involved in T cell activation, induces anergy among antigen-specific T cells and converts effector T cells into regulatory T cells [45, 54]. Blockade of this interaction was subsequently evaluated and the initial clinical trial demonstrated impressive tumor regression in various refractory tumor types, including RCC, melanoma, non-small cell lung cancer and colorectal cancer [55].

Immune checkpoint inhibition has quickly become a major focus of research over the past decade given its durable response rates and promising survival benefits in various malignancies. A number of inhibitors in the PD-1 pathway are currently being developed actively in clinical trials. These include nivolumab and pembrolizumab, which are PD-1 inhibitors, as well as atezolizumab, durvalumab and avelumab, which are PD-L1 inhibitors [56]. There are also anti-CTLA-4 antibodies that have been developed, including ipilimumab and tremelimumab [57]. Currently, multiple clinical trials studying the efficacy of these agents on mRCC are being conducted, with nivolumab being the only agent that is approved by the USFDA for the treatment of RCC [58].

**Figure 1:**
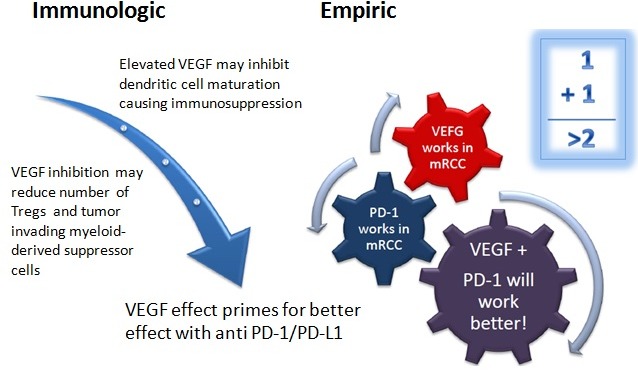
Schematic of the possible mechanism behind the synergy of anti-VEGF TKI's and anti PD-1/PD-L1 antibodies (Figure used with permission from Dr. Elizabeth Plimack, Fox Chase Cancer Center)

In addition, there are multiple ongoing trials investigating whether immunotherapy combinations, with each other or with targeted therapy, can yield additional benefit in mRCC. Chen and Mellman proposed a cancer-immunity cycle in which a series of stepwise events are needed in order for an anticancer immune response to lead to effective killing of cancer cells. Combining agents that target different parts of the cycle may lead to synergistic effects. For example, anti-CTLA-4 antibodies enhance priming and activation of antigen-specific T cells, while the blockade of the PD-1 pathway removes the inhibition of cancer cell killing by T cells [59]. This combination appears to provide clinical activity distinct from monotherapy alone in metastatic melanoma [60]. In addition, immunotherapy in combination with agents that enhance T cell trafficking and infiltration into the tumor bed, such as VEGF inhibitors, may also potentially provide additional clinical benefit not seen with either modality alone (Figure 1) [61–63].

Checkpoint inhibitors have their limitations in that a large proportion of patients do not respond to the treatments. In general, tumors with a higher mutational burden are associated with better response and durable clinical benefit [64]. The expression of PD-L1 also appears to correlate with elevated tumor-infiltrating lymphocytes and is associated with response to monoclonal antibodies targeting the PD-1/PD-L1 pathway [65]. Based on these principles, recent research have shown that increasing effector T cells tumor infiltration may improve the efficacy of PD-L1 checkpoint blockade [66].

As a class, checkpoint inhibitors are associated with a range of immune-related adverse events. The most common and typically earliest onset adverse reaction is dermatologic toxicity, which is usually managed by topical corticosteroid cream. Diarrhea/colitis is also common, but has a much higher incidence with CTLA-4 antibodies than antibodies targeting PD-1/PD-L1. Hepatotoxicity and endocrinopathy in the form of hypophysitis and hypothyroidism are also occasionally encountered. Other organs that may potentially be affected include lung, eye, kidney, pancreas, as well as neurologic or hematologic syndromes [67].

The following section describes the various checkpoint inhibitors that are being investigated in the management of mRCC (Table 1).

**Table 1:** Current ongoing clinical trials involving checkpoint inhibitors in mRCC

Primary Drug	Phase	Line	Malignancy	Arms	NCT number
Nivolumab	IV	2^nd^	RCC	Nivolumab	NCT02596035
Atezolizumab	III	1^st^	RCC	Atezolizumab + Bevacizumab Sunitinib	NCT02420821
Avelumab	III	1^st^	RCC	Avelumab + Axitinib Sunitinib	NCT02684006
Nivolumab + Ipilimumab	III	1^st^	RCC	Nivolumab + Ipilimumab Sunitinib	NCT02231749
Atezolizumab	II	1^st^	RCC	Atezolizumab Atezolizumab + Bevacizumab Sunitinib	NCT01984242
Ipilumumab	II	1^st^/2^nd^	RCC	Ipilimumab	NCT00057889
Nivolumab	II	1^st^	RCC	Nivolumab (pre- and post-op)	NCT02446860
Atezolizumab	I/II	2^nd^	Solid tumors	Atezolizumab + Varlilumab	NCT02543645
Pembrolizumab	I/II	1^st^	RCC	Pembrolizumab Pazopanib Pembrolizumab + Pazopanib	NCT02014636
Pembrolizumab	I/II	1^st^/2^nd^	RCC	Pembrolizumab + Bevacizumab	NCT02348008
Pembrolizumab	I/II	1^st^/2^nd^	RCC	Pembrolizumab + Vorinostat	NCT02619253
Pembrolizumab	I/II	2^nd^	Solid tumors	Pembrolizumab + Lenvatinib	NCT02501096
Pembrolizumab	I/II	2^nd^	Solid tumors	Pembrolizumab + Epacadostat	NCT02178722
Nivolumab	I/II	2^nd^	Solid tumors	Nivolumab + Varlilumab	NCT02335918
Atezolizumab	I	2^nd^	Solid tumors	Atezolizumab + CPI-444 CPI-444	NCT02655822
Avelumab	I	1^st^	RCC	Avelumab + Axitinib	NCT02493751
Durvalumab + AMP-514	I	2^nd^	Solid tumors	Durvalumab + AMP-514	NCT02118337
Durvalumab + Tremelimumab	I	2^nd^	Solid tumors	Durvalumab + Tremelimumab	NCT01975831
Ipilimumab	I	2^nd^	Solid tumors	Ipilimumab + MGA271	NCT02381314
Pembrolizumab	I	1^st^	RCC	Pembrolizumab + Axitinib	NCT02133742
Pembrolizumab	I	2^nd^	Solid tumors	Pembrolizumab + Ziv-Afilbercept	NCT02298959
Pembrolizumab	I	2^nd^	Solid tumors	Pembrolizumab + INCB039110 Pembrolizumab + INCB050465	NCT02646748
Pembrolizumab	I	2^nd^	Solid tumors	Pembrolizumab + MGA271	NCT02475213
Pembrolizumab + Ipilimumab	I	2^nd^	RCC, melanoma	Pembrolizumab Pembrolizumab + Ipilimumab Pembrolizumab + PEG IFN-α-2b	NCT02089685
Nivolumab	I	1^st^/2^nd^	RCC	Nivolumab + Sunitinib Nivolumab + Pazopanib Nivolumab + Ipilimumab	NCT01472081
Nivolumab	I	2^nd^	Solid tumors	Nivolumab + IFN-γ	NCT02614456
Nivolumab	N/A	1^st^/2^nd^	RCC	Nivolumab Nivolumab + Bevacizumab Nivolumab + Ipilimumab	NCT02210117

(Varlilumab = an anti-CD27 monoclonal antibody; Vorinostat = a histone deacetylase inhibitor; Epacadostat = an indoleamine 2,3-dioxygenase inhibitor; CPI-444 = an antagonist of the adenosine-A2A receptor; AMP-514 = an anti-PD-1 monoclonal antibody; MGA271 = an anti-B7-H3 monoclonal antibody; ICNB039110 = a JAK inhibitor with JAK1 selectivity; ICNB050465 = a PI3K-delta inhibitor)

### Nivolumab

Nivolumab (Opdivo^®^, Bristol-Myers Squibb, Princeton, NJ) is a fully human IgG4 anti-PD-1 immune checkpoint inhibitor monoclonal antibody. It has been studied extensively in various cancers, and has received approval by the USFDA for the treatment of mRCC, in addition to metastatic melanoma and squamous and non-squamous non-small cell lung cancer, and more recently, in Hodgkin Disease [68, 69].

Its initial development in renal cancer was as a phase II trial involving previously treated metastatic ccRCC patients. Nivolumab was dosed at 0.3, 2 and 10 mg/kg every 3 weeks, and was noted to produce ORR in 20-22% of patients with an OS of 18.2-25.5 months [70]. Given the promising data in this phase II study, a randomized, open-label, phase III study was developed comparing nivolumab to everolimus in patients who received prior treatment (Check Mate 025). 821 patients were randomly assigned 1:1 to receive either nivolumab 3 mg/kg intravenously every 2 weeks or everolimus 10 mg orally every day. The median OS were 25.0 months (95% confidence interval (CI), 21.8-not estimable) in the nivolumab group and 19.6 months (95% CI, 17.6-23.1), with the hazard ratio (HR) for death of 0.73 (98.5% CI, 0.57-0.93; *P* = 0.002) favoring the nivolumab group. The objective response rates also favored the nivolumab group (25% *vs*. 5%; *P* < 0.001), though the median PFS were similar (4.6 months *vs*. 4.4 months; HR 0.88; *P* = 0.11). The authors postulated that there might be a potential delayed benefit in PFS with nivolumab based on the late separation of the PFS curves and that PFS was not a surrogate for OS in this study. A benefit was observed with nivolumab irrespective of PD-L1 expression. Grade 3 or 4 treatment-related adverse events were less in the nivolumab group (19%) compared with the everolimus group (37%). Given improvement in efficacy and fewer treatment-related side effects compared to everolimus, nivolumab was established as a new standard of care in the management of advanced clear cell RCC in the second-line setting [58]. Nivolumab is under investigation as pre- and post-operative therapy in metastatic RCC (ADAPTeR) [NCT02446860] [71] and is also being studied in combination with other drugs [NCT01472081, NCT02231749, NCT02210117, NCT02335918, NCT02614456] [72–76].

### Pembrolizumab

Pembrolizumab (Keytruda^®^, Merck, Whitehouse Station, NJ) is a highly selective humanized IgG4 antibody that targets the PD-1 receptor and USFDA approved metastatic melanoma, squamous and non-squamous non-small cell lung cancer, and head and neck cancer [81]. It is currently being investigated for use in mRCC. Preliminary safety data from a recent phase I/II study (KEYNOTE-029) involving pembrolizumab plus ipilimumab or pegylated interferon alfa-2b (PEG-IFN) in patients with metastatic melanoma and RCC was recently reported [82]. There are also multiple ongoing studies evaluating pembrolizumab in combination with various drugs with different mechanisms [NCT02014636, NCT02133742, NCT02348008, NCT02089685, NCT02501096, NCT02619253, NCT02298959, NCT02646748, NCT02178722, NCT02475213] [83–92].

### Atezolizumab

Atezolizumab is a fully humanized monoclonal IgG1 antibody that targets PD-L1. It is being evaluated in multiple different cancers, including RCC. A recent phase Ia study involving atezolizumab in mRCC was recently reported. Of the 63 patients with clear cell RCC that were evaluable, median PFS was 5.6 months and median OS was 28.9 months. The objective response rate was 15% (18% in patients with > 1%, and 9% in those with < 1% PD-L1 expression) [93]. Several other trials involving atezolizumab are currently underway [NCT01984242, NCT02420821, NCT02543645, NCT02655822] [94–97].

### Avelumab

Avelumab (MSB0010718C) is a fully human anti-PD-L1 IgG1 monoclonal antibody that works by inhibiting PD-1/PD-L1 interactions while leaving PD-1/PD-L2 pathway intact. It may also induce antibody-dependent cell-mediated cytotoxicity (ADCC) by retaining a native Fc region. In a phase Ib study, avelumab was used in patients with refractory solid tumors and showed similar toxicity profiles compared to other PD-1 or PD-L1 inhibitors [98]. Two ongoing trials are evaluating avelumab in combination with axitinib [NCT02493751, NCT02684006] [99, 100].

### Durvalumab

Durvalumab (MEDI4736) is another human anti-PD-L1 IgG1 monoclonal antibody. It blocks PD-L1 binding to PD-1 and CD80, with no binding to PD-L2. ADCC and complement-dependent cytotoxicities are removed by an engineered triple mutation in the Fc domain. A Phase 1/2, multicenter, open-label study which evaluated the safety and clinical activity of the drug in patients with multiple solid tumor types such as non-small cell lung cancer noted very manageable safety profile [101]. There are ongoing trials evaluating durvalumab in combination with other drugs, including tremelimumab (a fully human monoclonal antibody against CTLA-4) [NCT01975831] [102] and MEDI0680 (AMP-514) (a humanized IgG4 monoclonal antibody against PD-1) [NCT02118337] [103] for patients with advanced malignancies including RCC.

### Ipilimumab

Ipilimumab (Yervoy^®^, Bristol-Myers Squibb, Princeton, NJ) is an anti-CTLA-4 IgG1 monoclonal antibody that is USFDA approved in melanoma [104]. It has been investigated as a single agent and in combination with nivolumab in metastatic melanoma, with the combination shown to be more effective albeit with significantly more toxicity [77]. This combination of nivolumab and ipilimumab is currently being investigated in mRCC. In a phase I study, patients were randomized into 2 groups with different dosing combinations followed by maintenance nivolumab until disease progression or unacceptable toxicity. Objective responses were noted in 15 of 44 patients (34%) and stable disease was seen in another 16 patients (36%) [78]. Further trials investigating ipilimumab alone [NCT00057889] [79] and in combination with other drugs are ongoing [NCT02231749, NCT02381314] [73, 80].

### Tremelimumab

Tremelimumab is another anti-CTLA-4 antibody that is actively being investigated in mRCC. Unlike ipilimumab, it is an IgG2 antibody. As discussed previously, it is currently being evaluated with durvalumab in the treatment of patients with mRCC [NCT01975831] [102].

### Vaccines

Vaccines are also under active investigation in RCC. They are aimed at treatment of the primary tumor rather than prevention, and uses the patient’s tumor cells or tumor-associated products for immune recognition. Clinical trials evaluating various vaccines have been conducted, although none has demonstrated an improvement in survival thus far. AGS-003 is a dendritic cell (DC) based vaccine in which mature DCs are co-electroporated with amplified tumor mRNA and synthetic CD40L RNA. CD40L expression on the DC surface is thought to cause T-cell activation by inducing co-stimulatory signals. A phase II study on 21 intermediate or poor-risk mRCC patients eligible for nephrectomy were treated by a combination therapy of AGS-003 with sunitinib. Median PFS and OS were 11.2 and 30.2 months respectively [105]. Based on these results, a phase III ADAPT study is currently underway, in which mRCC patients undergoing debulking nephrectomy are randomly assigned to sunitinib alone or sunitinib plus AGS-003 [NCT01582672] [106].

IMA-901 is a vaccine developed from multiple tumor-associated peptides (TUMAP) that are naturally presented in human cancer tissue. In a phase II trial utilizing the co-administration of cyclophosphamide (which reduces the inhibitory regulatory T cells), IMA-901 demonstrated a disease control rate of 31% at 6 months in patients previously treated with cytokine therapy, and 14% at 6 months in patients previously treated with tyrosine kinase inhibitors [107]. In a separate phase III trial, 339 patients were randomly assigned to receive sunitinib or to sunitinib plus IMA901 and GM-CSF. OS was not improved with the addition of the IMA901 vaccine compared with sunitinib alone [108].

TroVax (MVA-5T4) is a therapeutic vaccine targeting a glycosylated 5T4 antigen expressed on human placental trophoblasts and various human cancer cells. This particular tumor-associated antigen is overexpressed in most RCCs [109]. In a phase III TRIST trial, MVA-5T4 in combination with IFN-α, IL-2 or sunitinib as first-line mRCC therapy did not result in a significant increase in OS when compared to the arm without MVA-5T4 [110].

There are other ongoing trials involving DC-based vaccines. Some of the promising ones involve transduction of a fusion gene construct of GM-CSF and carbonic anhydrase IX into autologous DCs [NCT01826877] [111], pidilizumab (a PD-1 antibody) in combination with DC/RCC fusion cells [NCT01441765] [112], and DCs in combination with cytokine-induced killer cells [NCT00862303] [113].

### Adjuvant immunotherapy

Given the success and promising data involving checkpoint inhibition in metastatic RCC, it is reasonable to ask the question whether immunotherapy plays a role in the non-metastatic adjuvant setting. However, various randomized trials of adjuvant therapy involving tumor cells plus BCG, tumor cell vaccination, IFN-α, high-dose IL-2, or a combination of cytokines have not demonstrated survival benefit when compared with observation [114]. The role of checkpoint inhibition has not been investigated in the adjuvant setting. Therefore, at this time, observation is still the recommended approach in stage I-III RCC following nephrectomy.

### Immunotherapy in combination with radiotherapy

RCC is often considered to be a radio-resistant disease and the role of radiation is generally limited to palliative or local control. Preclinical data from the 1990’s from a RCC mouse model demonstrated the regression of lung metastases with radiation and an even greater degree of decrease when combined with IL-2 [115]. In addition, over the past decade, there is growing evidence that stereotactic radiotherapy may overcome resistance, although currently no randomized clinical trials have been done. Although the exact mechanism is not known, one hypothesis is that when radiotherapy is applied in a high-dose, few-fraction schedule, dendritic cells are recruited to the irradiated site, which adopt the tumor antigens and presented to cytotoxic T lymphocytes in the lymph nodes [116]. Currently, there is an ongoing clinical trial evaluating radiation therapy in combination with pembrolizumab for patients with recurrent or mRCC [NCT02318771] [117].

## CONCLUSION

The role of immunotherapy in the management of RCC has changed dramatically since the turn of the century. Previously, cytokine therapy involving IL-2 and IFN-α was the mainstay of mRCC treatment. However, benefit was only marginal considering the toxicities related to therapy. Over the past decade, significant progress has been made in the development of new immunotherapeutic agents in the form of checkpoint inhibition and vaccines, and these agents are gradually being incorporated in the treatment of mRCC and many other cancers, such as melanoma, non-small cell lung cancer, Hodgkin’s disease, and head and neck cancer. In mRCC, various checkpoint inhibitors are currently being investigated for use as a first-line agent, following recent USFDA approval of nivolumab in the second-line setting. As the use of immunotherapy becomes more widespread in oncology, many questions will certainly need to be addressed. For example, the long-term side effects and resistance mechanisms of these drugs in various tumors are important considerations. Optimizing treatment dose and schedule, investigating biomarkers that may affect disease outcomes, and evaluating the combination of different treatment modalities are also critical in maximizing the potential of immunotherapy [67]. Further research and experience in this field will allow us to better identify strategies in utilizing immunotherapeutic agents not just in RCC, but also in many other malignancies.

It is now quite clear that immunotherapy is here to stay and has begun to impact the clinical outcome of patients with mRCC. Further enhancements to this efficacy paradigm can be achieved with the use of combination therapies, including with other checkpoint inhibitors and with anti-VEGF TKI. As alluded to earlier in this review, there is strong pre-clinical rationale for combining immunotherapy with anti-VEGF inhibitors. The latter, in particular, have been shown to reduce myeloid derived stem cells (MDSC) that are crucial in tumor-induced immune suppression. Furthermore, development and clinical implementation of predictive biomarkers will be crucial to the use of immunotherapy. Preliminary research, especially the expression of PD-L1 by IHC in the tumor cells, has so far not lived up to the promise of a predictive biomarker [58]. One of the criticisms has been that the tumor samples that are being evaluated are derived from primary site and in the treatment naive setting, which are bound to be immunologically different from cancer samples that have already been exposed to prior therapies. Furthermore, while the knowledge derived from the Cancer Genome Atlas (TCGA) effort is likely to provide a lot more insight into the biology of the tumors, it underperforms on the immunotherapy front, since the major host immune response lies in the stromal cells, which was not part of the TCGA evaluation. Other potential biomarkers include the preexistence of CD8+ tumor infiltrating lymphocytes (TIL), though these have been directly associated with anti PD-1 therapy only in melanoma and MSI high colorectal cancer [118, 119]. This needs to be explored further in renal cancer.

In this review, we have sought to highlight the entire field of clinical development of immune based therapy for mRCC, and hope that the reader will find this piece of work useful as a summary and as a source of reference. We also recognize that it is a highly dynamic and evolving field, and the pace of development is astounding and encouraging. The use of immune based therapy for this disease, while decades old (in the form of INF and IL-2), offers new promise with the targeting of the checkpoint pathways. The latter field is still in its infancy stage, with tremendous potential for the future. We believe that a major paradigm shift in RCC management will only occur once we can increase the cure rates of surgically managed patients by finding a drug that will improve survival rates in the adjuvant setting. Such trials using TKI and anti PD-1/PD-L1 agents are in progress, and the results are eagerly awaited.
